# Excitability properties of mouse and human skeletal muscle fibres compared by muscle velocity recovery cycles

**DOI:** 10.1016/j.nmd.2022.02.011

**Published:** 2022-02-26

**Authors:** K. J. Suetterlin, R. Männikkö, E. Matthews, L. Greensmith, M.G. Hanna, H. Bostock, S.V. Tan

**Affiliations:** 1 Department of Neuromuscular Diseases, UCL Queen Square Institute of Neurology, London, UK; 2 AGE Research Group, NIHR Newcastle Biomedical Research Centre, Newcastle upon Tyne Hospitals NHS Foundation Trust and Newcastle University, Newcastle upon Tyne, UK; 3 Atkinson Morley Neuromuscular Centre, Department of Neurology, St Georges University Hospitals NHS Foundation Trust, London, UK; 4 Department of Neurology and Clinical Neurophysiology, Guy's & St Thomas' NHS Foundation Trust and Institute of Psychiatry, Psychology & Neuroscience, Division of Neuroscience, King's College London, UK

**Keywords:** Muscle Velocity Recovery Cycles (MVRCs), Excitability, Membrane, Ion Channel, Channelopathies, Non-dystrophic Myotonia, Periodic Paralysis, Mouse models, Translation

## Abstract

Mouse models of skeletal muscle channelopathies are not phenocopies of human disease. In some cases (e.g., Myotonia Congenita) the phenotype is much more severe, whilst in others (e.g. Hypokalaemic periodic paralysis) rodent physiology is protective. This suggests a species’ difference in muscle excitability properties. In humans these can be measured indirectly by the post-impulse changes in conduction velocity, using Muscle Velocity Recovery Cycles (MVRCs). We performed MVRCs in mice and compared their muscle excitability properties with humans. Mouse Tibialis Anterior MVRCs (n=70) have only one phase of supernormality (increased conduction velocity), which is smaller in magnitude (p=9×10^-21^), and shorter in duration (p=3×10^-24^) than human (n=26). This abbreviated supernormality is followed by a period of late subnormality (reduced velocity) in mice, which overlaps in time with the late supernormality seen in human MVRCs. The period of late subnormality suggests increased t-tubule Na^+^/K^+^-pump activity. The subnormal phase in mice was converted to supernormality by blocking chloride channels, suggesting relatively higher chloride conductance in mouse. Our findings help explain discrepancies in phenotype between mice and humans with skeletal muscle channelopathies and potentially other neuromuscular disorders. MVRCs are a valuable new tool to compare *in vivo* muscle membrane properties between species and will allow further dissection of the molecular mechanisms regulating muscle excitability.

## Abbreviations

9AC: 9 anthracene carboxylic acid
α2-pump: alpha-2 isoform of the Na^+^/K^+^-ATPase
ClC-1: skeletal muscle voltage-gated chloride channel
ESN: earlysupernormality
ISI: interstimulus interval
Kir: inward rectifier potassium channel
LSN: late supernormality
MC: myotonia congenita
MRRP: muscle relative refractory period
MVRC: muscle velocity recovery cycle
PP: periodic paralysis
TA: tibialis anterior

## 1 Introduction

Animal models of disease allow exploration of physiological functions and systemic interactions between organs [1]. Mice have become the preferred model of disease, due to their relative ease of genetic manipulation, similarities in biochemistry and physiology and low cost [2]. However, translation of findings from animal models of neuromuscular disease to human patients has been poor [2,3]. Understanding how and why the neuromuscular functions differ between the species will improve the value of the mouse as a model organism and may even indicate potential protective mechanisms that inform novel therapies.

Skeletal muscle channelopathies are rare disorders, caused by mutations in genes encoding skeletal muscle ion channels that result in over- or under-excitability of the muscle. The classic skeletal muscle channelopathies present clinically with myotonia or periodic paralysis (PP), respectively. Non-dystrophic myotonias are caused by loss-of-function mutations of the skeletal muscle chloride channel ClC-1 or by gain-of-function mutations of the skeletal muscle sodium channel Na_V_1.4 [4]. Myotonia related to altered ClC-1 function is also observed in Myotonic Dystrophy type 1 and 2. Periodic paralyses (PP) are caused by gain-of-function mutations in either Na_v_1.4 or the skeletal muscle calcium channel Ca_V_1.1 or loss of function in Kir2.1 [4]. Mutations in ion channels have also been reported in association with congenital myopathy[5,6] and congenital myasthenia [7–9].

The species difference in channelopathy presentation is exemplified by mouse models of myotonia congenita (MC) [10] and hypokalaemic PP [11,12]. In contrast to humans with recessive MC, who are considered non-dystrophic and believed to have normal lifespan, adr mice with biallelic loss-of-function in chloride channels, as in MC, exhibit reduced life span, low body weight and significant muscle atrophy. This is despite similar reductions in sarcolemmal chloride conductance between adr mice and humans with recessive MC [13,14].

Conversely, whilst spontaneous attacks of weakness are one of the defining clinical features of hypokalaemic PP in humans, the two transgenic mouse models of hypokalaemic PP have locomotor behaviour indistinguishable from controls [11,12]. This apparent resistance to spontaneous paralytic attacks was also observed in an acquired (potassium-deficient) rat model of periodic paralysis [15]. As changes in muscle ion channels show different consequences in mice and humans, we suspected that the intrinsic muscle excitability properties may differ between the species.

To obtain information about muscle membrane potential and ion channels, we have used the recently established method of muscle velocity recovery cycles (MVRCs) [16]. The velocity of muscle action potentials increases during the negative afterpotential following an impulse, and this provides a sensitive indication of changes in resting membrane potential [17]. Single-fibre recordings have demonstrated the validity of this approach to understanding muscle membrane dysfunction in muscular dystrophy and denervation [18] but have proved too variable and technically challenging for clinical use. The use of multi-fibre recordings [16] has overcome this limitation, and multi-fibre MVRCS using 1-5 conditioning stimuli have been validated as a repeatable technique [19,20], that reveals consistent evidence of membrane depolarization in patients with chronic renal failure [21] and critical illness myopathy [22,23], and evidence of dysfunction of specific ion channels in patients with mytonia congenita [24], sodium channel myotonias [25], Andersen-Tawil syndrome [26] and periodic paralyses [27].

We have previously shown that MVRC recording is technically feasible in mice and is also sensitive enough to detect changes in muscle excitability properties due to genetic ion channel dysfunction [28]. Here we compare MVRCs recorded *in vivo* from human and mouse muscles and describe pharmacological experiments to help account for the marked species differences in intrinsic membrane properties. These differences may help explain some of the differences in channelopathy presentation described above.

## 2 Methods

### 2.1 Animals

C57/BL J6 mice were used: 34 recordings were from male tibialis anterior (TA) muscle and 36 recordings were from female TA. The mean age of animal was 28 ± 16.2 weeks, and the range was 13 to 77 weeks. We have previously shown that mouse TA MVRCs with up to 5 conditioning stimuli do not change significantly with age [28] . Mice were fed *ad libitum* and housed according to home office guidelines. Experiments were carried out under licence from the UK Home Office (Scientific Procedures Act 1986) and following approval by the UCL Institute of Neurology Animal Welfare Ethical Review Panel.

### 2.2 Healthy Human Volunteers

Human recordings were performed on the TA of 10 healthy male volunteers (age 48±12 years) and 16 healthy female volunteers (age 42±13 years) as previously described [24,26,29]. Informed written consent was obtained from all subjects according to the Declaration of Helsinki. The study was approved by St Thomas Hospital, London, UK and University College London research ethics committees (10/H0802/6).

### 2.3 Mouse Muscle Velocity Recovery Cycle (MVRC) recording *in vivo*

In contrast to humans, an anaesthetic is required to perform MVRCs in mice. For the first few recordings, when developing the MVRC technique chloral hydrate administered via intraperitoneal injection was used. However, we changed to inhaled anaesthesia as this can be adjusted and maintained without requiring additional intraperitoneal injection, which on occasion disturbed recording electrodes. However, inhalational anaesthetics have been shown to modulate Na_v_ channels *in vitro* and isoflurane is known to reduce cortical excitability (Pelosi *et al*., 2001). There is no such report for chloral hydrate. There was no apparent difference in the morphology or parameters of the initial mouse MVRCs recorded using intraperitoneal chloral hydrate or the subsequent MVRCs recording using inhaled isoflurane anaesthesia. This is in keeping with the finding that there was no difference in mouse peripheral nerve excitability measurements recorded under the influence of inhaled isoflurane or intraperitoneal injection of chloral hydrate (Boërio, Greensmith and Bostock, 2011) and the finding that sevoflurane had no effect on the recovery cycle of human cortical neurons (Burke *et al*., 2000)

**Figure 1:**
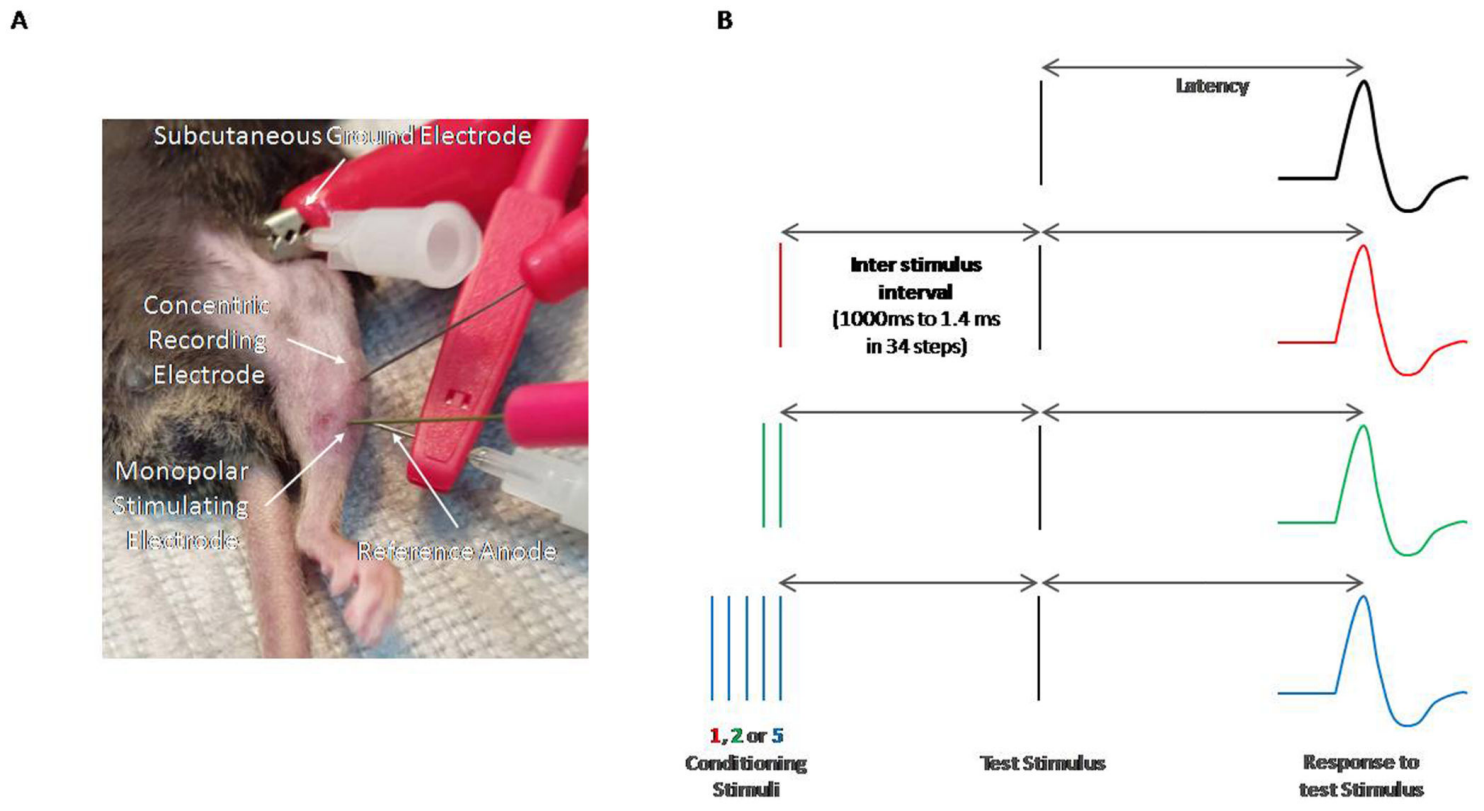
Experimental setup for MVRCs in mouse TA muscle. A monopolar stimulating needle electrode (28G TECA, Viasys Healthcare Madison, Wisconsin) was inserted into the distal muscle. A reference anode was inserted above the monopolar stimulating electrode on the lateral edge of the muscle. The reference anode consisted of a 27G hollow bore disposable steel needle attached to reference anode lead with crocodile clip. Stimuli consisting of 0.05ms rectangular current pulses were delivered. Muscle activity was recorded with a concentric needle electrode (disposable 30G concentric EMG needle, TECA) inserted into the proximal end of the muscle. A ground electrode was inserted under the skin in the axilla. The ground electrode consisted of a 27G hollow bore disposable steel needle that was bent to make it easier to insert under the skin and attached to crocodile clip on the ground cable. **B.** A schematic showing the stimulation and recording protocol is shown. One, 2 or 5 conditioning stimuli are given at 34 different time intervals between 1.4 and 1000ms before a test stimulus. The latency of the CMAP peak in response to the test stimulus is measured. This latency is then plotted as a percentage change as compared to the test stimulus alone (see Fig 2).

After induction, the mouse was placed on its back on a heat mat and anaesthesia maintained via a nose cone (Fig.1**)**. MVRCs were performed as described previously [28]. The signal was amplified at a gain of 1000, filtered with bandwidth 50Hz to 2kHz and digitised (NI DAQ) using a sampling rate of 20 kHz. The electrodes were adjusted to obtain a stable negative peak response with a stimulus of 3 -10 mA. Stimulation and recording were controlled by QTRAC software using the M3REC3.QRP protocol. Surface temperature over the muscle was recorded at the end of the recording using an infra-red thermometer. MVRCs were recorded with 1, 2 and 5 conditioning stimuli, all separated by 10 ms intervals. Test stimuli were delivered every 2 s. The inter-stimulus interval between the last conditioning stimulus and the test stimulus varied from 1000 to 1.4 ms in 34 steps in an approximately geometric series (specifically 1000, 900, 800, 700, 600, 500, 450, 400, 350, 300, 260, 220, 180, 140, 110, 89, 71, 56, 45, 35, 28, 22, 18, 14, 11, 8.9, 7.1, 5.6, 4.5, 3.5, 2.8, 2.2 and 1.8 ms, Fig 1B) [16,24].

A conditioned stimulus refers to a test stimulus preceded by either 1, 2 or 5 conditioning stimuli at one of the 34 different interstimulus intervals. An unconditioned stimulus refers to a test stimulus alone. The time from test stimulus to the peak of compound muscle action potential response is always measured and referred to as latency (Fig 1B). The latency change compares response to a conditioned test stimulus with response to an unconditioned test stimulus at each of the 34 different interstimulus intervals (Fig 1B).

### 2.4 MVRCs with ClC-1 inhibition

Baseline MVRCs were performed (as described above). Afterwards intraperitoneal injection of 9 anthracene carboxylic acid (9AC) was administered with MVRC recording electrodes still *in situ*. The dose of 9AC used was either 5 mg/kg or 30 mg/kg (as described for an *in vivo* rat model of myotonia [30]). A minimum of 10 minutes after injection, MVRCs were repeated on the same TA before being performed on the contralateral TA. For this reason, when all recordings were successful there could be more recordings post injection than pre injection from the same animal. All recordings were completed within 60 minutes of 9AC administration.

### 2.5 Statistical Analysis

To determine statistical significance Welch or Welch rank test was performed depending on normality (Liliefor’s test). As multiple parameters were compared an increased threshold for statistical significance of p≤0.01 was applied.

## 3 Results

### 3.1 Muscle Velocity Recovery Cycles (MVRCs)

**Figure 2:**
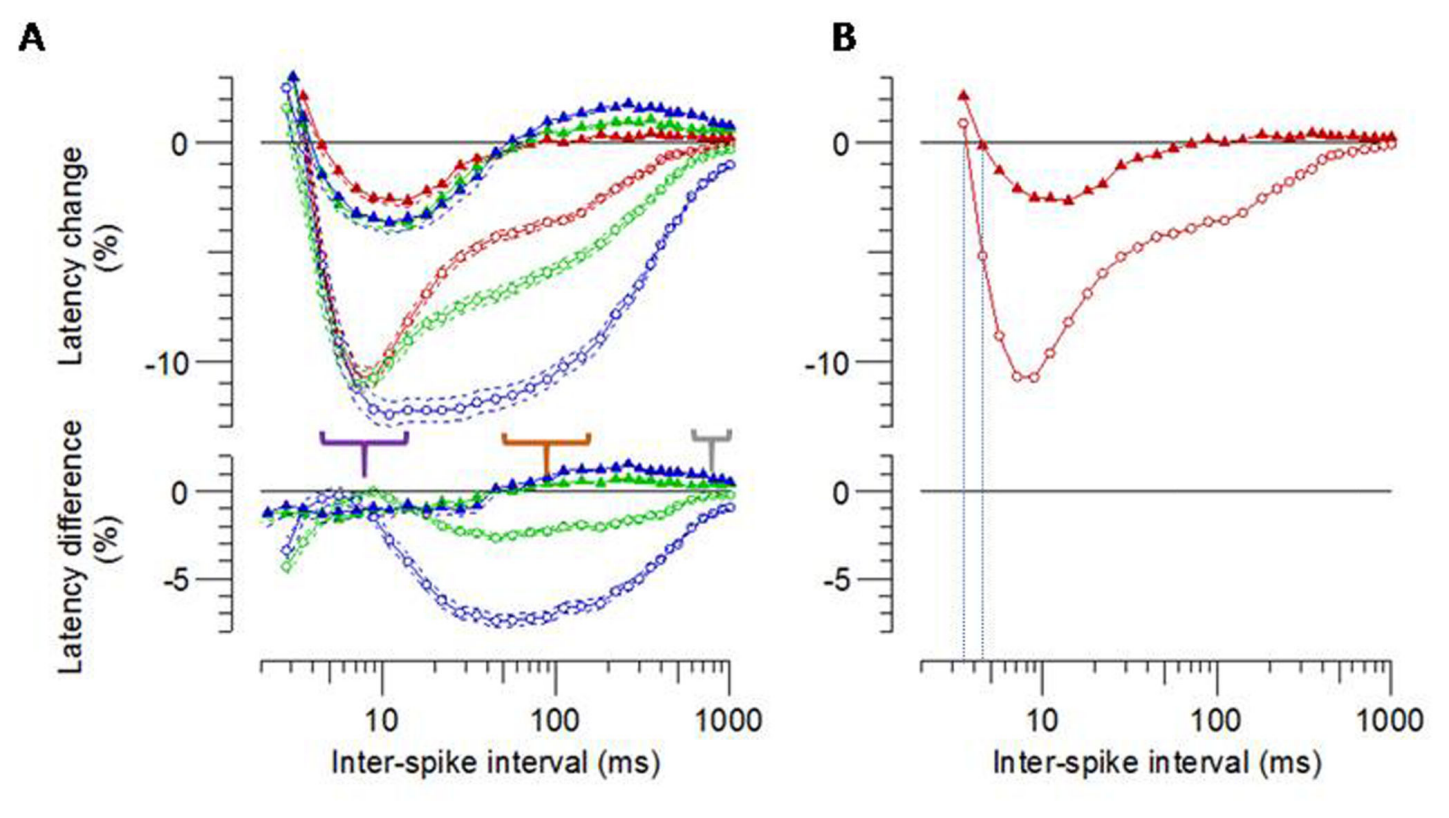
Comparison of MVRCs from humans and mice. **A.** Comparison of MVRCs in response to 1 (red), 2 (green) and 5 (blue) conditioning stimuli from humans (n=26, open circles) and mouse TA (n=70, filled triangles). The purple bracket delineates the period of early supernormality, the orange bracket the period of late supernormality and the grey bracket residual supernormality. **B.** Data is the same as in A but limited to MVRCs in response to 1 conditioning stimulus to demonstrate the increased Muscle Relative Refractory Period (MRRP) in mouse compared to human TA. MRRP is the point at which the latency change between conditioned and unconditioned stimuli is 0% and represents the end of the relative refractory period of the excitability recovery cycle. The dashed lines mark the interstimulus interval corresponding to the MRRP. Data are mean±SEM

Human MVRCs have two phases of supernormality (Fig 2A). The first peaks before an interstimulus interval (ISI) of 15 ms (Table 1, Fig 2A, purple bracket). This is referred to as ‘early supernormality’ (ESN) [16] and has been proposed to reflect the effect of the depolarising afterpotential. The second phase, which typically increases in magnitude with larger numbers of conditioning stimuli, has been proposed to reflect the depolarising effect of potassium accumulation in the t-tubules, and is referred to as ‘late supernormality ‘(LSN) because it is usually maximal at an ISI of 50 to 150 ms [16] (Fig 2A orange bracket, Table 1) . The late supernormality gradually declines over about 1s, although in response to 5 conditioning stimuli there is usually some residual supernormality (RSN) in human MVRCs at an inter-stimulus interval of 1000 ms (Fig 2A grey bracket, Table 1) [24].

The morphology of mouse MVRCs showed clear differences from human recordings (Fig 2, Table 1). Firstly, mouse MVRCs have only one phase of supernormality. This single phase of supernormality is smaller (ESN mouse 3.33 ± 0.29% vs human 11.19 ± 0.44%, p=9.2×10^-21^)and peaks later (10.78ms ± 0.36 mouse vs 7.98ms ± 0.23 human, p=7.1×10^-9^) than the first phase of supernormality in human MVRCs and ends at an inter-stimulus interval of about 50 ms (mean 53.6 ±5.1), equivalent in timing to the early part of the late supernormal period in humans. Secondly, this single period of supernormality in mouse MVRCs is followed by a period of late *sub*normality that gradually reduces towards baseline at an ISI of about 1000 ms (Table 1, Fig 2). Late subnormality has not been reported for human or pig MVRCs [16,31,32]. Thirdly, in mice, increasing the number of conditioning stimuli has a small and uniform effect across the MVRC whilst in humans, increasing the number of conditioning stimuli has a larger and more disproportionate effect on late supernormality (Fig 2). Furthermore, in contrast to the increase in magnitude of the late supernormality seen in humans [16,31,32], increasing the number of conditioning stimuli increased the degree of late *sub*normality in mouse, i.e. 5 conditioning stimuli resulted in both increased supernormality and increased late subnormality in mice (Table 1, Fig 2A). Finally, the muscle relative refractory period (MRRP) (time interval at which there is no difference between a conditioned and unconditioned stimulus) was significantly longer in mouse TA compared to human TA MVRCs (Fig 2B, Table 1. p=0.001).

### 3.2 MVRCs with ClC-1 Inhibition

**Figure 3:**
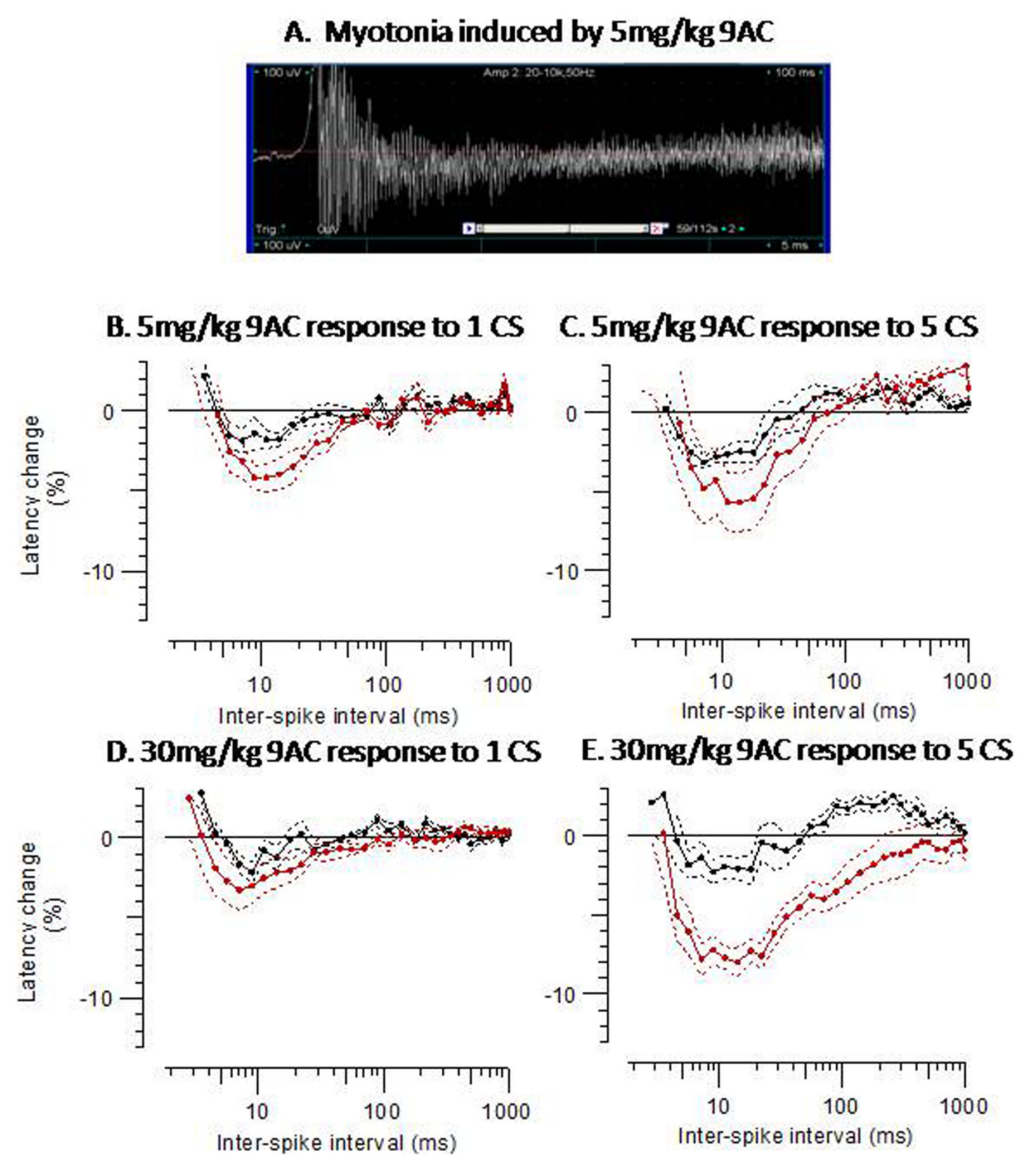
The effect of 9-Anthracene Carboxylic Acid (9AC) on mouse MVRCs. **A**. Mouse TA EMG recording showing myotonia induced by 5mg/kg 9AC. **B**. Mouse TA MVRCs in response to 1 conditioning stimulus pre (black, n=7) and post (red, n=6) 5mg/kg 9AC. **C**. Mouse TA MVRCs in response to 5 conditioning stimuli pre (black, n=7) and post (red, n=6) 5mg/kg 9AC. **D**. Mouse TA MVRCs in response to 1 conditioning stimulus pre (black, n=4) and post (red, n=8) 30mg/kg 9AC. **E**. Mouse TA MVRCs in response to 5 CS pre (black, n=4) and post (red, n=8) 30mg/kg 9AC. Data are mean±SEM

A dose of 5mg/kg intraperitoneal 9AC was sufficient to induce clinical and electrical myotonia (Fig 3A). This was evident by approximately 6 minutes post injection. MVRCs performed at least 10 minutes after intraperitoneal injection of 5mg/kg 9AC appeared to increase the amplitude and duration of supernormality but maintained overall mouse MVRC morphology (i.e., a single phase of supernormality that is followed by subnormality).

Moreover, there were no significant differences in MVRC parameters compared to MVRCs from the same animals recorded prior to intraperitoneal injection of 9AC (Fig 3B & C).

Increasing the dose of 9AC to one established for a rat model of myotonia congenita (30mg/kg) [30] increased the duration of supernormality to a single stimulus (p=0.004), removed late *sub*normality and induced late *super*normality in mouse TA (Fig 3D, Table 2). The presence of 30mg/kg 9AC augmented the effect of conditioning stimuli, especially for the late supernormality that was now present (Fig 3E, Table 2). There was a trend towards a reduced MRRP with 30mg/kg 9AC but this effect was not statistically significant (Fig 3, Table 2).

## 4 Discussion

This study was undertaken to test the hypothesis that there are differences in intrinsic membrane properties between mouse and human skeletal muscle. MVRCs demonstrate that mouse muscle membrane properties are very different from those in humans. Therefore, it is not surprising that the clinical presentations of genetically identical channelopathies also differ between the two species. We review the ionic membrane conductance that underlies early and late supernormality and how this differs between mice and humans. We then consider possible physiological reasons why these differences may be necessary and the relevance of these differences to the use of mice as models of neuromuscular diseases in general and muscle channelopathies in particular.

### 4.1 The Ionic Basis of Early and Late Supernormality

The biophysical basis of early and late supernormality has been discussed in detail (Z'Graggen & Bostock, 2009). As first proposed by Frank for frog muscle [33], the rapid repolarization phase of the action potential ends with the membrane slightly depolarized (as influx of sodium ions exceeds efflux of potassium ions), and this negative afterpotential decays passively. As in myelinated nerve, it results in an increase in excitability and conduction velocity [17,34]. Similarly, there is a late afterpotential, first described in frog muscle [35]. The late afterpotential summates with trains of impulses and associated potassium accumulation in the t-tubule system. The late afterpotential correlates with the late phase of supernormality seen in MVRCs [16].

### 4.2 How do muscle membranes in mice differ from those in humans?

The most striking differences between mouse and human MVRCs are the replacement of impulse-dependent late *super*normality by impulse-dependent late *sub*normality and the reduced effect of conditioning stimuli in mouse muscle. Both observations point to species’ differences in the buffering or removal of activity-dependent increases in t-tubule potassium.

In rats and presumably also in mice, the effect of potassium accumulation in the t-tubules is countered by the t-system chloride conductance [36]. The fact that healthy human muscle shows marked activity-dependent supernormality but healthy mouse muscle exhibits this only when the ClC-1 channel is blocked suggests a relatively larger contribution of ClC-1 current in limiting membrane potential depolarization upon t-tubule potassium accumulation in mice. When we blocked ClC-1 channels with 9AC (Fig 3), the conduction velocity became sensitive to the number of conditioning stimuli and there was clear late supernormality and even residual supernormality. The effect of ClC-1 blockade on late supernormality also provides indirect evidence of ClC-1 localisation to the t-tubules as the late afterpotential, with which late supernormality correlates, is abolished with de-tubulation [35].

Like our mouse recordings with 9AC, MVRCs from humans with myotonia due to reduced ClC-1 conductance exhibit increased supernormality with the predominant effect on late and residual supernormality [24]. As for the mice, the sensitivity to conditioning stimuli is also increased in patients with MC and the increase in late and residual supernormality is most marked in response to 5 conditioning stimuli [24].

We have not found direct measurements of both sarcolemmal membrane conductance *and* chloride conductance in mice or humans, but in isolated EDL muscles from female Wistar rats the chloride conductance constituted approximately 90% of total resting membrane conductance (1314 ±72 μS/cm^2^, and 1458 ± 70 μS/cm^2^ respectively) (Pedersen et al 2009). In human muscle, combined blockade of chloride and sodium conductance resulted in a 69.3% reduction of resting membrane conductance in human abdominal muscle (Riisager et al 2016), suggesting that the percentage contribution of chloride conductance to total resting membrane conductance was at least 20% lower in human abdominal muscle compared with rat EDL. In addition, total resting membrane conductance of human abdominal muscle (427 ± 16 μS/cm^2^) appeared significantly lower than in rodent muscle (about 30% of that measured in rat EDL). If mouse muscle has similar properties to rat, then it is possible that, not just as a proportion of total resting membrane conductance but also in absolute terms, skeletal muscle chloride conductance may be much larger in mouse than in humans. If this is true, then the late supernormality seen in large mammals might be shorted out by relatively greater t-tubular chloride conductance in rats and mice.

However, it was intriguing that we required such high doses of 9AC - more than that required to trigger myotonia - to see late and residual supernormality in mouse MVRCs. One possible reason for this discrepancy between the presence of clinical myotonia with 5mg/kg 9AC, but no significant difference on MVRCs with 5 conditioning stimuli is that physiological activation of muscle will involve much longer trains of action potentials than the 5 conditioning stimuli delivered during MVRCs. The fact that there is clinical myotonia with 5mg/kg 9AC suggests that the dose is sufficient to alter membrane excitability, but, in contrast to humans with MC [24], 5 conditioning stimuli is not sufficient to cause significant change on mouse MVRCs. This may be due to more effective t-tubule potassium reuptake in mouse muscle such that chloride channel blockade must be near complete for the effect of 5 conditioning stimuli on mouse MVRCs to be seen.

Two mechanisms have been described in mouse muscle for the removal of t-tubule potassium: inward rectifier (Kir) potassium currents [37] and the alpha-2 isoform of the Na^+^/K^+^-ATPase sodium pump (α2-pump) [38]. Of these only the α2-pump, with its 3:2 Na^+^:K^+^ stoichiometry, generates a net hyperpolarizing current while moving potassium intracellularly. For this reason, and because it has been shown to be strongly activated by t-tubule potassium almost immediately following an action potential [38], the α2-pump is the obvious candidate for generating the impulse-dependent late subnormality. The hyperpolarization would then assist further potassium removal by the Kir channels. Phenotyping the skeletal muscle of α2-pump knock out mice provides some support for this hypothesis. These mice are apparently normal under basal conditions but show significantly reduced exercise capacity when challenged to run [39]. The authors conclude that the α2-pump “is regulated by muscle use and enables working skeletal muscles to maintain contraction and resist fatigue.” [39]. Unfortunately, an *in vivo* dose for selective blockade of the α2-pump in skeletal muscle is not established. Developing the MVRC technique so it can be performed on isolated muscles *ex vivo* would enable this hypothesis to be tested as micromolar ouabain could be used to selectively inhibit the α2-pump [38].

### 4.3 Why should muscle function be different in mice and humans?

Muscle fibres in mice appear structurally similar to those in humans, with diametres of about 50μm, similar sliding filament contractile apparatus, with resting sarcomere length close to 2.5 μm [40,41], juxtaposed to a similar t-tubule system. However, the observation that 30mg/kg 9AC made mouse MVRCs appear more human-like argues against these mouse-human differences being the direct effect of muscle geometry but instead a functional adaption to it.

The laboratory mouse shares ^~^99% of its genes with humans and for many years transgenic mice have been used successfully to determine the function of skeletal muscle proteins [42,43]. Yet, when it comes to using mice as models for neuromuscular disease, there are limitations [44–47]. This may in part be due to biomechanical differences in muscle function during walking [46], but the most obvious difference between mice and humans, is that they differ in size and weight by over three orders of magnitude – and the ‘physiological clock’ of smaller animals ticks faster: smaller animals have shorter lives, they grow up and reproduce more quickly, their hearts beat faster, and their movements are more rapid [48–50].

Animals of similar shape differing by a factor of 1000 in size have muscles that differ little in force per unit area, and differ little in running speed, but the smaller animal has to move its limbs 10 times more rapidly to achieve this [49]. If humans tried to contract TA muscles 10 times more rapidly (and were not prevented by inertial forces from doing so) they would soon stop because of muscle fatigue, due mainly to potassium-induced membrane depolarization [51]. It follows that a mouse TA muscle with the same membrane properties as human TA would fatigue very rapidly, due to potassium accumulation in the t-tubules, so the membrane properties must be different in smaller animals. As A.V. Hill argued: ‘the intrinsic speed of a muscle has to vary inversely with length. The chemical engineer, therefore, in designing a muscle, had to plan its enzymes and proteins so that the speed of its reactions was adjusted to the dimensions of the body into which it fitted..’ [49].

As discussed in section 4.2, the subnormality observed on mouse MVRC suggests increased activity of the α-2 pump in mouse compared to human skeletal muscle. There is data to support a relatively greater Na/K-ATPase activity in smaller mammals [50,52], although the α-2 pump, to our knowledge, has not been specifically examined. BMR scales with surface area or mass^2/3^, so that for a 1000-fold difference in weight, BMR per unit mass is 10 times higher for the smaller animal [53]. The higher BMR per unit mass is associated with greater Na/K-ATPase activity required to maintain transmembrane gradients, since smaller animals have leakier membranes due to a higher content of polyunsaturated phospholipids [50,52]. Leakier sarcolemma may also contribute to the reduced depolarizing afterpotential and early supernormality seen in mouse MVRCs (Fig 2).

One limitation of this study is that we were not able to record *in vivo* from a slow-twitch predominantly oxidative mouse muscle. Mice have a far higher proportion of fast twitch glycolytic fibres than humans and they also have myosin heavy chain isoform type IIb fibres which are not present in human muscle[54]. Soleus is one of the few oxidative mouse muscles and has no type IIb fibres [54]. However, as soleus lies deep to the gastrocnemius and does not induce a specific, easily identifiable movement on contraction, it was impossible to be certain that we were recording from soleus *in vivo*. Determining the contribution of differences in fibre-type to species difference in MVRC profile will be a priority for future work. This is particular important given the reported differences in ClC-1 conductance, Na_v_ expression and resting membrane potential between fast and slow-twitch muscle fibres [55].

### 4.4 Relevance to mice as models of neuromuscular disease

Our demonstration of marked differences in muscle excitability properties between mice and humans provides some insight into the phenotype differences between mice and humans with skeletal muscle channelopathies [10–12,56,57], and perhaps also other mouse models of neuromuscular disease [2,45,58,59].

The association of recessive myotonia congenita in mice, but not humans, with reduced body weight, muscle atrophy and a reduced life span [10] is in keeping with a more fundamental role of ClC-1 channels in mouse skeletal muscle, as indicated by our findings with 9-anthracene carboxylic acid. Reduced ability of ClC-1 knockout animals to counter the effect of potassium accumulation likely provides a large metabolic burden on the mice by increasing reliance on energy-dependent α2-pump activity to maintain t-tubule potassium homeostasis. A similar reduction in lifespan has not been reported for transgenic mouse models with myotonia secondary to mutation in Na_v_1.4 [56,57] but male Draggen mice with Na_v_1.4 myotonia do show higher energy expenditure and reduced total fat mass compared to their wild-type siblings [56]. There was no sex difference reported for transgenic models of MC – both male and female mice exhibited reduced body weight and life span [10].

However, it was the observation of phenotype difference and relative resistance to periodic paralysis attacks that initially inspired this work. This study does not allow us to comment definitively on the mechanism of resistance to spontaneous attacks in periodic paralysis mice. However, the inferred greater activity of the α2-pump in mice is particularly interesting given that selective block of isolated muscle from mice with the Na_V_1.4 R669H mutation for Hypokalaemic PP with 1μM ouabain prevented recovery of force following exposure to hypokalaemia and lowered the threshold for hypokalaemia-induced weakness to occur [12]. Understanding the precise mechanisms involved requires further work but is, we believe worth pursuing, as it should improve translation of studies using transgenic mice as a model of periodic paralysis and may even highlight novel therapeutic options for people with periodic paralysis.

MVRCs will be an effective tool for studying alterations to muscle physiology caused by a wide variety of muscle disorders, since membrane excitability properties are altered not just in conditions directly affecting muscle ion channels, but also as a consequence of pathology affecting muscle metabolism/energy supply, protein kinases and other interlinked processes [21–23,60–63]. The fact that MVRCs can be performed in both mice and humans with the same condition should facilitate effective translation of findings from mouse models to human subjects. In addition, where a defined effect on skeletal muscle excitability (e.g. membrane depolarisation) is observed, MVRCs may also be a useful mechanism to screen drugs for neuromuscular disorders and/or skeletal muscle side effects.

### 4.5 Conclusion

In summary, we demonstrate significant differences in mouse and human skeletal muscle excitability. Our data proposes that in mouse muscle, higher relative functional expression of the of the ClC-1 chloride channel and α2-pump contribute to reduced sensitivity to activity-dependent t-tubule potassium accumulation. This is likely an adaptation to a higher rate of muscle contraction in small animals. Our findings provide initial insights into the differences between mouse and human muscle physiology. A better understanding of these differences will enable more robust translation of data obtained from studies of mouse models of human neuromuscular disease. MVRCs are a valuable new tool that enables comparison of muscle membrane properties between species and will allow further characterisation of the molecular mechanisms regulating muscle excitability *in vivo*.

## Figures and Tables

**Table 1:** Human and Mouse Muscle Velocity Recovery Cycle Measurements (MVRCs) compared using parameters described for human MVRCs.

Excitability Measures	Mean ± SE (n)		P for Welch rank test (non parametric)
	Human TA	Mouse TA	Human Vs Mouse
MRRP	3.70 ± 0.12 (26)	4.79 ± 0.23 (67)	p=0.00128^*^
ESN (%)	11.19 ± 0.44 (26)	3.33 ± 0.29 (67)	p=9.21×10^-21^^*^
ESN@(ms)	7.98 ± 0.23 (26)	10.78 ± 0.36 (67)	p=7.08×10^-9^^*^
SNEnd (ms)	745.3 ± 39.5 (26)	53.55 ± 5.12(66)	p=6.97×10^-23^^*^
5ESN (%)	13.01 ± 0.54 (26)	4.90 ± 0.51 (62)	p=1.21×10^-15^^*^
LSN (%)	3.68 ± 0.17 (26)	0.013 ± 0.066 (70)	p=2.61×10^-24^^*^
2XLSN (%)	2.25 ± 0.15 (26)	-0.323 ± 0.075 (70)	p=3.12^-^×10^23^^*^
5XLSN (%)	7.03 ± 0.31 (26)	-0.83 ± 0.15 (70)	p=2.62×10^-24^^*^
RSN (%)	0.125 ± 0.037 (26)	-0.248 ± 0.044 (68)	p=8.90×10^-9^^*^
5XRSN (%)	0.986 ± 0.081 (26)	-0.568 ± 0.068 (68)	p=1.15×10^-21^^*^

TA, Tibialis Anterior; MRRP, muscle relative refractory period; ESN, early supernormality (up to 15 ms); ESN@, interstimulus interval for maximum ESN; SNEnd, time supernormal period to 1 conditioning stimulus ends, 5ESN, early supernormality after 5 conditioning stimuli; LSN, late supernormality (50–150 ms); 2XLSN extra supernormality after 2 conditioning stimuli compared with 1 conditioning stimulus; 5XLSN, extra supernormality after 5 conditioning stimuli compared with 1 conditioning stimulus; RSN, residual supernormality (900–1000 ms); 5XRSN, extra residual supernormality after 5 conditioning stimuli.

* p<0.01

**Table 2:** Mouse TA Muscle Velocity Recovery Cycle Measurements (MVRCs) pre and post 30mg/kg 9 Anthracene Carboxylic Acid Administration compared using parameters described for human MVRCs.

Excitability Measures	Mean ± SE (n)		P for Welch rank test (nonparametric)
	Mouse TA Pre 9AC	Mouse TA Post 9AC	Pre Vs post 9AC
MRRP	4.565 ± 0.388(4)	3.568 ± 0.517(6)	p=0.18159
ESN (%)	2.245 ± 0.619(4)	4.736 ± 1.07(7)	p=0.09406
ESN@(ms)	8.45 ± 0.45(4)	8.057 ± 1.25(7)	p=0.32082
SNEnd (ms)	26.32 ± 7.55 (4)	213.2 ± 92.9 (8)	p=0.004^*^
5ESN (%)	2.965 ± 0.513(4)	9.003 ± 0.837(8)	p=0.0003^*^
LSN (%)	-0.5475 ± 0.0981(4)	0.3513 ± 0.194(8)	p=0.0003^*^
2XLSN (%)	-0.205 ± 0.249(4)	0.855 ± 0.785(8)	p=0.39552
5XLSN (%)	-0.8475 ± 0.338(4)	2.964 ± 1.03(8)	p=0.00634^*^
RSN (%)	0.07 ± 0.122(4)	-0.34 ± 0.241(7)	p=0.12528
5XRSN (%)	-0.5125 ± 0.351(4)	0.7229 ± 0.726(7)	p=0.24414
Latency(ms)	1.757 ± 0.147(4)	2.041 ± 0.0797(8)	p=0.23724

TA, Tibialis Anterior; MRRP, muscle relative refractory period; ESN, early supernormality (up to 15 ms); ESN@, interstimulus interval for maximum ESN; SNEnd, time supernormal period to 1 conditioning stimulus ends, 5ESN, early supernormality after 5 conditioning stimuli; LSN, late supernormality (50–150 ms); 2XLSN extra supernormality after 2 conditioning stimuli compared with 1 conditioning stimulus; 5XLSN, extra supernormality after 5 conditioning stimuli compared with 1 conditioning stimulus; RSN, residual supernormality (900–1000 ms); 5XRSN, extra residual supernormality after 5 conditioning stimuli.

* p<0.01
